# Fabrication of Oxygen Vacancy-Rich WO_3_ Porous Thin Film by Sputter Deposition for Ultrasensitive Mustard-Gas Simulants Sensor

**DOI:** 10.3390/s25103049

**Published:** 2025-05-12

**Authors:** Haizhen Li, Cancan Yan, Jun Shen, Shuai Liu, Qingyu Ma, Yongchao Zheng

**Affiliations:** 1 School of Materials Science and Engineering, University of Jinan, Jinan 250022, China; 202111100031@stu.ujn.edu.cn (H.L.); mse_maqy@ujn.edu.cn (Q.M.); 2 State Key Laboratory of Chemistry for NBC Hazards Protection, Beijing 102205, China; ccy805905145@163.com (C.Y.); s17852097397@163.com (J.S.); 202232809@stumail.nwu.edu.cn (S.L.)

**Keywords:** magnetron sputtering, sulfur mustard simulants, tungsten trioxide, porous film, oxygen vacancy

## Abstract

Exposure to sulfur mustard can result in severe injury or even fatalities in humans. Therefore, the development of reliable and high-performance sensors for detecting sulfur mustard is critical. Herein, WO_3_ thin films are prepared as sulfur mustard simulant (e.g., 2-chloroethyl ethylsulfide, 2-CEES) sensing materials using sputter deposition followed by high-temperature annealing. The 2-CEES gas sensors fabricated via WO_3_ porous films realize high-performance detection of 2-CEES at 260 °C with an impressive detection limit (15 ppb), fast response (58 s), long-term stability, and good selectivity. Through systematic optimization of deposition and annealing parameters, WO_3_ porous thin films with tailored oxygen vacancy concentrations were prepared, facilitating device fabrication. This approach provides an effective strategy for the batch production of miniaturized devices enabling real-time monitoring of vesicant agents.

## 1. Introduction

Sulfur mustard (HD, 2-bis (2-chloroethyl) sulfide) is a highly toxic vesicant chemical warfare agent that can induce severe skin blistering, damage mucous membranes and the respiratory tract, and may cause fatal outcomes even at low concentrations of exposure [1,2]. Due to its extremely low Immediately Dangerous to Life and Health (IDLH) threshold (100 ppb), the development of low-cost real-time detection techniques for HD has become critically imperative, addressing both public safety assurance and national defense requirements [2]. Given the high toxicity of HD, 2-chloroethyl ethyl sulfide (2-CEES), which has similar physicochemical properties to HD but low toxicity, is often used as a substitute in laboratories [3]. Among various detection methods, chemiresistive gas sensors based on metal oxide semiconductors (MOS), such as ZnO [4,5], SnO_2_ [6], Fe_2_O_3_ [7]_,_ and WO_3_ [3,8], etc., have gained significant attention due to their operational simplicity, cost-effectiveness, easy miniaturization, and proven capability in detecting HD and its simulants like 2-CEES. However, MOS-based 2-CEES gas sensors are typically fabricated using powder drop-coating and spin-coating techniques, which present significant challenges in maintaining sensing device consistency [9,10].

The sputtering deposition method serves as a critical technology in fabricating MOS gas sensors, facilitating the integration of nanostructured MOS materials with MEMS-based sensing platforms [11]. The sputter-deposited MOS film demonstrates high quality, uniformity, and superior substrate adhesion [12,13]. Furthermore, the film’s properties (crystallinity, thickness, and oxygen vacancy, etc.), which are closely associated with gas sensing performance, can be precisely tuned through the adjustment of sputtering parameters (oxygen flow, sputtering time, and chamber pressure, etc.) and annealing temperature [14,15,16,17,18]. Therefore, engineering MOS films with porous structures and enhanced surface oxygen vacancies via magnetron sputtering followed by controlled annealing may enable high-performance gas sensors for HD/CEES detection.

With this in mind, we employed reactive DC magnetron sputtering to deposit phase-controlled WO_3_ films on silicon substrates, as WO_3_ is an attractive n-type semiconductor material with a tunable band gap (2.6–3.0 eV) which can achieve remarkable sensing responses in detecting hazardous compounds like NO_2_ and 2-CEES [3,19,20]. Through systematic optimization of deposition and annealing parameters, we engineered WO_3_ porous thin films with tailored oxygen vacancy concentrations, which critically enhance sensing performance toward 2-CEES at 260 °C. The resulting sensor can achieve remarkable sensitivity even at ultra-low 2-CEES concentrations of 15 ppb. This strategy creates favorable conditions for the batch fabrication of miniaturized devices, enabling real-time detection of vesicant agents.

## 2. Materials and Methods

Materials: The high-purity W (99.99%), Ti (99.99%), and Au targets (99.99%) were purchased from Zhongnuo New Material Technology Co., Ltd., Beijing, China. Si/SiO_2_ substrates (500 ± 10 μm) were purchased from Kaihua County Huake Silicon Materials Sales Department (Quzhou, China). 2-CEES and dimethyl methylphosphonate (DMMP) were obtained from Sigma-Aldrich (Louis, MO, USA). Ethanol (AR, 99.5%) and acetone (AR, 99%) were sourced from Sinopharm Group Chemical Reagent Co., Ltd. (Beijing, China). Ammonia, hydrogen sulfide, and nitrogen dioxide were supplied by Anxing Tailong Gas Chemical Co., Ltd. (Beijing, China). Ultrapure water was self-prepared in the laboratory. All reagents used in the experiment were of analytical grade and were not further purified.

**Figure 1:**
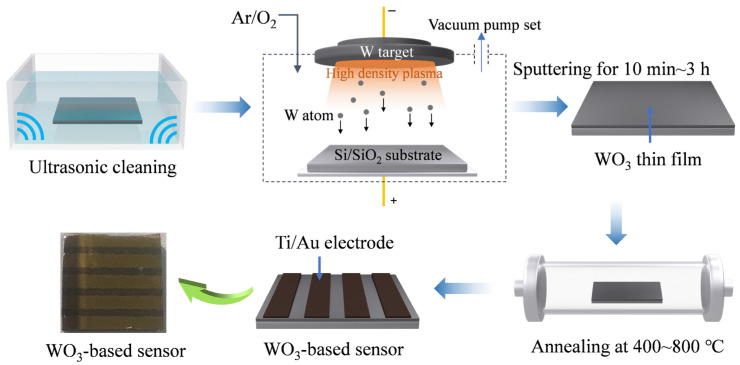
Schematic of gas sensor based on a WO_3_ thin film fabricated via magnetron sputtering.

Preparation of WO_3_ thin film: In this work, WO_3_ films were deposited on Si/SiO_2_ substrates (0.7 × 0.7 cm) with a thickness of ~500 μm using DC magnetron sputtering followed by high-temperature annealing. The structural and morphological properties of the films were optimized by precisely controlling the sputtering time and annealing temperature, as illustrated in Figure 1. Firstly, the Si/SiO_2_ substrates were sequentially subjected to ultrasonic cleaning in ethanol and deionized water to eliminate surface impurities. Subsequently, the cleaned substrates were transferred to the magnetron sputtering chamber and evacuated to achieve a high vacuum of 1.5 × 10^−4^ Pa. Then, oxygen and argon gases were introduced, and the flow rates were adjusted to maintain a ratio of 20:60 while keeping the total pressure at 1.0 Pa. Under a rotational speed of 8 r/min and a DC power of 100 W, the target underwent pre-sputtering for 5 min to remove surface contaminants. Following this, the sputtering time was controlled within a range from 10 min to 3 h to deposit WO_3_ films of varying thicknesses (~114 nm to ~3.0 μm). Finally, the deposited samples were placed in a tube furnace and annealed under an air atmosphere. The annealing temperature was set within a range from 400 to 800 °C with a heating rate of 5 °C/min, and the annealing process lasted for 4 h to refine the microstructure and optimize the physical and chemical characteristics of the films. WO_3_ films prepared under different conditions were systematically named in the format of WO_3_-sputtering time-annealing temperature (°C), for example, WO_3_-3 h-400.

Gas sensor fabrication and measurement: Ti/Au interdigitated electrode (IDE) was deposited on the WO_3_ thin film through a mask (0.5 mm spacing) using the magnetron sputtering technique. Initially, the Ti electrode with a thickness of ~40 nm was deposited under the following process conditions: the Ar flow rate was maintained at 60 sccm, the total pressure was controlled at 1.0 Pa, and the substrate was rotated at a speed of 8 r/min during deposition. Prior to the deposition, a pre-sputtering process was conducted at 50 W DC power for 5 min to remove surface contaminants. The actual deposition of the Ti electrode was then carried out for 60 s. Subsequently, the Au electrode with a thickness of ~40 nm was deposited using the same process parameters. While other conditions remained unchanged, the pre-sputtering time for the Au electrode was adjusted to 3 min, and the deposition time was extended to 80 s. The schematic of the device after electrode deposition is shown in Figure 1.

All gas-sensing properties of the WO_3_-based sensors were evaluated in dry air using an custom-designed dynamic gas-sensing detection system (Figure S1). The working temperature of the sensors was precisely controlled by a ceramic heating plate integrated at the bottom of the test chamber. 2-CEES gas was quantified by gas chromatography, and its concentration was determined using a standard curve (Figure S2), as described in our previous publication [8]. The response value (R) is defined as R = R_a_/R_g_ (R_a_ and R_g_ were the resistances of the sensors in air and the target gases, respectively). The real-time resistance of the sensors was monitored using a multimeter (model 2450, Keithley Instruments Inc., Cleveland, OH, USA).

Characterizations: X-ray powder diffractions (XRD) were conducted using the SmartLab XRD instrument (Rigaku, Tokyo, Japan) with a Cu Kα radiation source (λ = 0.15418 nm). The scanning rate was set at 2°/min. The microstructure and surface morphology of the films were analyzed using scanning electron microscopy (SEM) with the Nova NanoSEM 430 instrument (FEI, Hillsboro, OR, USA). X-ray photoelectron spectroscopy (XPS) analysis was performed using a XPS spectrometer (Thermo Fisher Scientific Inc., Waltham, MA, USA) equipped with a monochromatic Al Kα X-ray source. The binding energy calibration was referenced to sp^3^ hybridized carbon (C-C), with the C1s peak set at 284.8 eV. Atomic force microscopy (AFM) characterization was carried out using the Bruker Dimension Icon system (Bruker Corporation, Billerica, MA, USA). Gas chromatography (GC), which was performed on a Shimadzu GC-2014C spectrometer (Shimadzu Corporation, Kyoto, Japan), was used for the quantitative analysis of 2-CEES.

Computational details: First-principles calculations based on density functional theory (DFT) were performed to investigate WO_3_ using a plane-wave-based Vienna Ab initio Simulation Package (VASP) [21,22]. Structural relaxations and static energy calculations were conducted using the generalized gradient approximation (GGA) and the Perdew–Burke–Ernzerhof (PBE) functional [23]. The plane-wave basis cutoff energy was fixed at 400 eV for all atoms to ensure computational accuracy. Ionic positions were fully relaxed with an energy convergence criterion of 10^−6^ eV/atom and a force tolerance limited to 0.05 eV/Å per atom. Brillouin zone integration was carried out using a γ-centered Monkhorst–Pack scheme with a 3 × 3 × 1 K-point grid [24]. The WO_3_ (002) surface was identified based on transmission electron microscopy analysis, and a 2 × 2 supercell was constructed. Utilizing the optimized geometric structure, calculations were performed for the density of states (DOS), electronic band structure, and charge density differences. Molecular and charge density graphics were generated with VESTA [25].

## 3. Results

**Figure 2:**
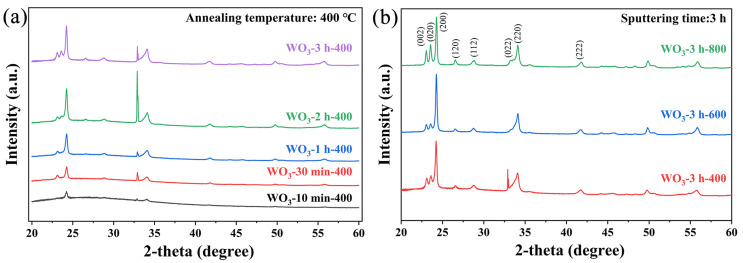
(**a**) XRD patterns of WO_3_ thin films annealed at 400 °C with different sputtering times, (**b**) XRD patterns of WO_3_ thin films deposited with a sputtering time of 3 h and annealed at different temperatures.

Amorphous WO_3_ was deposited directly on Si/SiO_2_ substrates via magnetron sputtering and then annealed in air to yield WO_3_ thin films. Powder XRD revealed that no clear diffraction peaks were observed in the patterns of the WO_3_-3 h-unannealed samples, indicating that the unannealed WO_3_ thin films were structurally amorphous (Figure S3). Conversely, the annealed WO_3_ thin films displayed good crystallinity, and their XRD patterns (Figure 2a) were consistent with standard monoclinic WO_3_, with three strong peaks at 24.4°, 32.9°, and 34.1° corresponding to the (200), (022), and (220) crystal planes, respectively [26]. As the sputtering time increased, the intensities of the diffraction peaks representing the (002) and (020) crystal planes were gradually enhanced, and the peak shapes sharpened, indicating that the grain size increased and the crystallinity improved [27]. When the sputtering time was kept constant, the crystallinity of the WO_3_ thin films varied significantly with different annealing temperatures (Figure 2b). The relatively broad peak shapes and several weak characteristic peaks in the pattern of WO_3_-3 h-400 indicated that the sample could still contain amorphous WO_3_ or smaller grains. As the annealing temperature increased to 600 and 800 °C, the diffraction peaks were clearly enhanced and sharpened, indicating that the grain size increased and the crystallinity of the film was enhanced [28]. Based on the Scherrer equation, the grain size of the WO_3_ film gradually increased as the annealing temperature increased, and the average grain sizes of WO_3_-3 h-400, WO_3_-3 h-600, and WO_3_-3 h-800 were 12.1, 16.6, and 19.9 nm, respectively.

**Figure 3:**
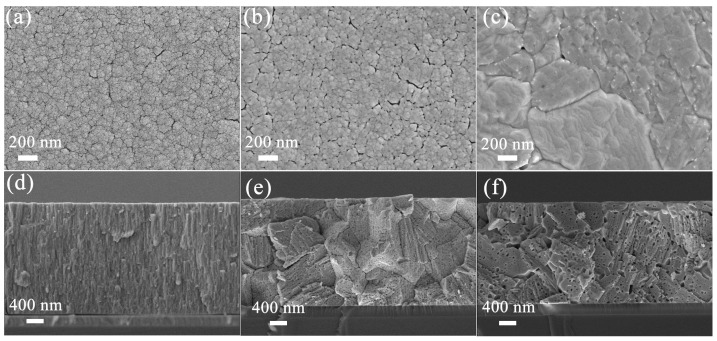
SEM images of WO_3_ thin film with a sputtering time of 3 h at different annealing temperatures: (**a**) 400 °C; (**b**) 600 °C; (**c**) 800 °C. (**d**–**f**) Cross-sectional SEM images of WO_3_ thin film annealed at 400–800 °C, respectively.

The results of SEM revealed that a distinct WO_3_ thin film formed on the Si/SiO_2_ substrate (Figure 3 and Figure S4). When the annealing temperature was 400 °C, the overall surface of the WO_3_ film was uniform, and a small number of fine cracks could be observed (Figure 3a). In addition, the lower annealing temperature led to a less dense film with obvious pores and interfacial defects [12], resulting in a porous structure (Figure 3a,b). When the annealing temperature was gradually increased to 800 °C, the sizes of the WO_3_ particles increased significantly, film densification was further enhanced, and the specific surface area decreased accordingly (Figure 3c). The corresponding cross-sections indicated that the porosity of the film interface gradually decreased as the annealing temperature increased, resulting in grains that were denser and better defined (Figure 3d–f).

**Figure 4:**
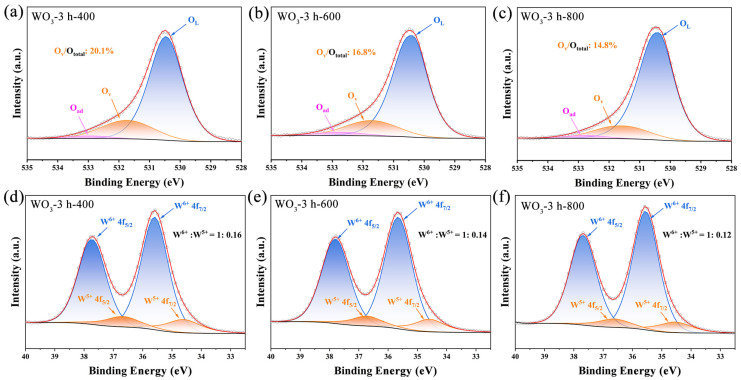
(**a**–**c**) High-resolution O 1s XPS spectra and (**d**–**f**) W 4f XPS spectra of WO_3_-3 h-400, WO_3_-3 h-600, and WO_3_-3 h-800, respectively.

The chemical states and surface compositions of WO_3_-3 h-400, WO_3_-3 h-600, and WO_3_-3 h-800 prepared with different annealing temperatures were further analyzed using XPS analysis. As shown in Figure 4a–c, the O 1s spectra of the samples could be deconvoluted into three characteristic peaks at ~530.4, ~531.7, and ~532.7 eV, which corresponded to lattice oxygen (O_L_), oxygen vacancy (O_V_), and adsorbed oxygen (O_ad_), respectively [29]. In the W 4f spectra (Figure 4d–f), two distinctive characteristic peaks of the WO_3_ films were observed at ~34.5 and ~36.6 eV, and they were attributed to the W 4f_7/2_ and W 4f_5/2_ orbitals of W^6+^, respectively. Conversely, the other two smaller peaks were located at ~35.6 and ~37.7 eV corresponding to the W 4f_7/2_ and W 4f_5/2_ orbitals of W^5+^, respectively [30].

**Figure 5:**
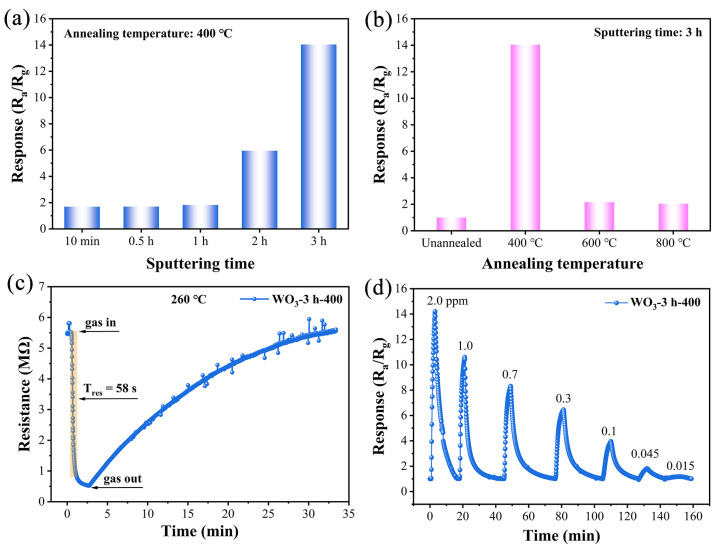
(**a**) Response values of WO_3_ thin films with different sputtering times to 2.0 ppm 2-CEES at 260 °C. (**b**) Response values of WO_3_-based sensors annealed at different temperatures to 2.0 ppm 2-CEES 260 °C. (**c**) Dynamic response–recovery curve of the WO_3_-3 h-400-based sensor to 1.0 ppm 2-CEES at 260 °C. (**d**) The dynamic response curve of the WO_3_-3 h-400-based sensor to 2-CEES in the concentration range of 0.015–2.0 ppm at 260 °C.

Based on the results of structural characterization, we prepared a series of gas sensors using WO_3_ thin-film materials via the modulation of the sputtering time and annealing temperature. Their sensing performances with respect to 2-CEES were tested using our custom-designed gas sensing detection system (Figure S1). As shown in Figure 5a, the response values (1.69, 1.70, 1.82, 5.95, and 14.04) of the WO_3_-based sensors prepared with different sputtering times to 2.0 ppm 2-CEES at an operating temperature of 260 °C improved significantly with increasing sputtering time. Hence, properly extending the sputtering time could improve the response performance of the sensor. When the sputtering time was 3 h, the response values of the samples annealed at 400 °C were considerably higher than those of the samples unannealed and annealed at 600 and 800 °C, and thus, 400 °C was the optimal annealing temperature (Figure 5b). Therefore, the WO_3_ thin films prepared with a sputtering time of 3 h and an annealing temperature of 400 °C exhibited the optimal sensing performances for 2-CEES. To reveal the effect of working temperature on the sensing performance of the WO_3_-3 h-400-based sensor, the working temperature-dependent response values were tested (Figure S5). As can be seen, the sensor exhibited its maximum response value of 10.6 to 1.0 ppm 2-CEES at 260 °C. Thus, 260 °C was chosen as the optimal working temperature. The dynamic response–recovery curves observed at 260 °C revealed that the resistance of the WO_3_-3 h-400-based sensor exposed to 1.0 ppm 2-CEES could rapidly decrease from 5.5 to 0.52 MΩ in 58 s (Figure 5c). Simultaneously, it could be completely recovered to the initial state, indicating that the sensor displayed good reversibility and a relatively rapid response. The dynamic response curve of the sensor over the 2-CEES concentration range 0.015–2.0 ppm indicated that the response values increased as the 2-CEES concentration increased. The response value still reached 1.18 at 0.015 ppm 2-CEES and thus, the lowest practical detection limit of the sensor was as low as 15 ppb, revealing an excellent low-concentration detection capacity.

**Figure 6:**
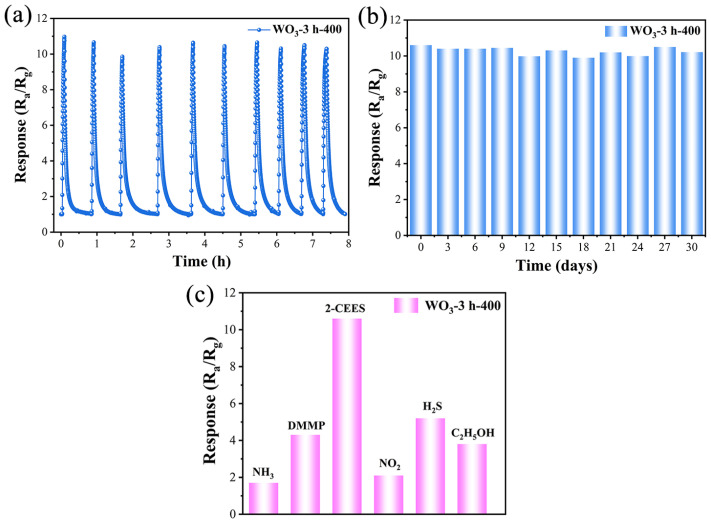
(**a**) The repeatability of the WO_3_-3 h-400-based sensor toward 1.0 ppm 2-CEES at 260 °C. (**b**) Long-term stability of the WO_3_-3 h-400-based sensor toward 1.0 ppm 2-CEES during 30 days at 260 °C. (**c**) The selectivity of the WO_3_-3 h-400-based sensor toward 1.0 ppm 2-CEES and five other different gases at 260 °C.

The repeatability, stability, and selectivity of the WO_3_-3 h-400-based sensor were further evaluated. Firstly, the response of the WO_3_-3 h-400-based sensor to 1.0 ppm 2-CEES was investigated over 10 consecutive cycles at 260 °C. The response values were consistently approximately 10.6, with a fluctuation of <2%, and the sensor exhibited excellent response–recovery characteristics, indicating that it displayed a good repeatability (Figure 6a). The results of a long-term stability study for 30 days under the same conditions revealed that the response value of the sensor did not change significantly over an extended time, and the stable detection performance could be maintained (Figure 6b). In addition, we investigated the selectivity of the WO_3_-3 h-400-based sensor for 2-CEES at 260 °C with interfering gases, including sarin simulants (DMMP) and other common volatile organic gases. The response value of the sensor to 1.0 ppm 2-CEES was more than twice as high as the values to other gases at identical concentrations (Figure 6c). In particular, the high selectivity of the sensor between 2-CEES and DMMP enables it to effectively discriminate between sulfur mustard and nerve agents, which is critical in detecting HD in practical applications. Above all, the WO_3_-3 h-400-based sensor shows its distinct ppb level sensing performance toward 2-CEES, which is comparable to, or even outperforms, most of the reported MOS-based 2-CEES gas sensors (Table S1).

## 4. Discussion

In this study, WO_3_-based sensors were prepared by modulating the sputtering time and annealing temperature, and the WO_3_-3 h-400-based sensor displayed the optimal sensing performance for 2-CEES. Under ambient air, the electrons within WO_3_ are trapped by the oxygen in the air, generating reactive oxygen species, such as O_2_^−^, O^−^, and O^2−^, on the surface of the film. These reactive oxygen species mostly occur as O^−^ ions at 260 °C [31]. The sensing of 2-CEES can be summarized as follows: When 2-CEES is adsorbed on a WO_3_ film at 260 °C, it first decomposes into •SCH_2_CH_3_ and ClCH_2_CH_2_• radicals, which are adsorbed onto the Lewis acid sites of the film via chlorine and sulfur atoms [32]. During adsorption, the radicals act as electron donors and interact with the Lewis acid sites [7]. Throughout the reaction, the captured free electrons are released into the conduction band of WO_3_, leading to a decrease in the resistance of the sensor.

**Figure 7:**
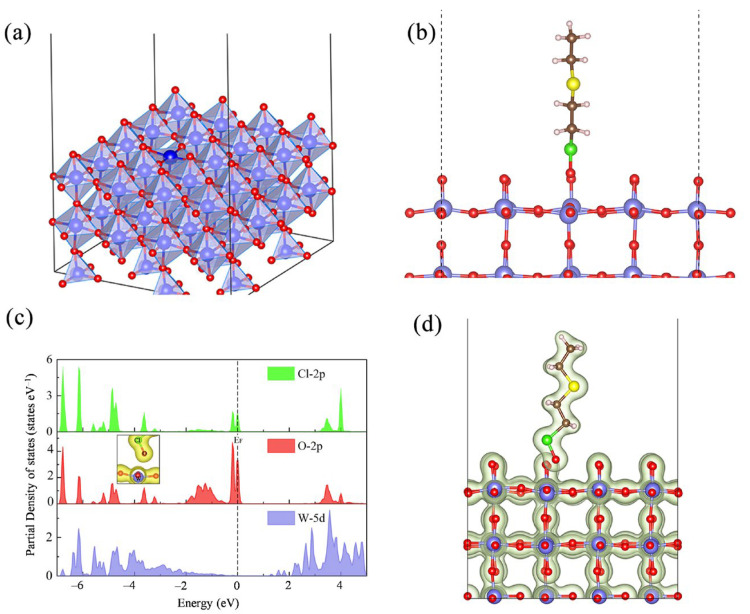
(**a**) Crystal structures of WO_3_, (**b**) models of the adsorbed 2-CEES on WO_3_, (**c**) partial density of states, and (**d**) charge densities of the 2-CEES on WO_3_. W (purple), O (red), Cl (green), C (brown), H (light pink), and S (yellow).

To understand the excellent sensing performance of the WO_3_-based sensors for 2-CEES in detail, density functional theory calculations were conducted to confirm the mode of the interaction between 2-CEES and WO_3_. A five-atom layer of WO_3_ was used as a model to simulate the WO_3_ thin-film material (Figure 7a). The adsorption behavior of 2-CEES on the WO_3_ surface was investigated, and the results revealed that 2-CEES generally bound to the prepared WO_3_ surface via Cl–O bonding, with an adsorption energy of –1.86 eV (Figure 7b). Meanwhile, significant orbital hybridization occurred between the Cl 2p, O 2p, and W 5d atoms within the WO_3_ model (Figure 7c), and the partial charge densities of the WO_3_ film and 2-CEES clearly overlapped (Figure 7d). Therefore, 2-CEES could effectively adsorb on the surface of WO_3_ and exhibit a high reactivity, which facilitated the high sensitivity and rapid responses of the WO_3_-based sensors.

**Figure 8:**
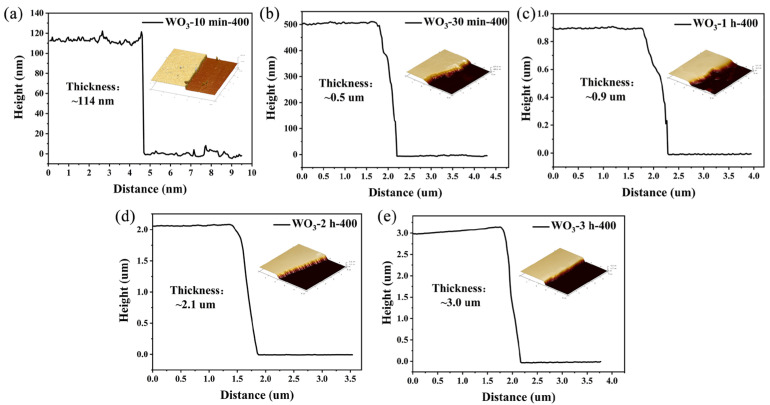
AFM images of WO_3_ thin films at different sputtering times: (**a**) 10 min; (**b**) 30 min; (**c**) 1 h; (**d**) 2 h; (**e**) 3 h.

Based on the results of material characterization and the gas-sensing studies, the gas-sensing properties of the WO_3_ thin films were significantly affected by their sputtering times and annealing temperatures. After annealing at 400 °C, the response values to 2-CEES at 260 °C of the WO_3_ thin films increased with the prolongation of the sputtering time. This could be attributed to the synergistic effect of several key factors. Firstly, the film thickness increased from 114 nm to 3.0 μm with increasing sputtering time, displaying a linear growth trend (Figure 8 and Figure S6). The thicker film exhibited increased surface roughness and the formation of porous structures (Figure S7), thereby enhancing the specific surface area and providing more active sites for gas adsorption/desorption reactions [33,34]. Secondly, the thickness of the film directly affected the structure of the carrier depletion layer of the material. The depletion layer of the thinner film could occupy most of the bulk phase region, resulting in a limited scope for changes in the carrier concentration. Conversely, a thicker film could generate a larger range of resistance changes, thus enhancing the response amplitude of the sensor [35]. In addition, an increase in the film thickness was generally accompanied by increases in grain size growth and crystalline quality. Highly crystalline WO_3_ films exhibited superior degrees of carrier transport and lower levels of scattering at their grain boundaries, contributing to the sensitivities of their gas-sensitive responses [36].

When the sputtering time was held constant, the response values of the WO_3_ films to 2-CEES decreased significantly with increasing annealing temperature, which could be attributed to changes in the microstructures and surface chemical properties of the films [37,38]. Firstly, the results of XRD and SEM revealed that a higher annealing temperature significantly promoted grain growth, which densified the WO_3_ films and reduced their porosities. This microstructural evolution crucially influenced the sensing performance, as gas sensing relies on the diffusion of the target gas molecules within the porous structures of the films and their ad−/desorption interactions with the active sites [39]. The narrowing of the pore size could limit the effective penetration of gas molecules, thus reducing the gas-sensing response [40]. In addition, the high densities of the films could reduce their specific surface areas and the accessibility of the active sites, further weakening their gas-sensitive properties [41]. Secondly, the results of XPS indicated that the different annealing temperatures significantly affected the oxygen vacancy content of the WO_3_ films. As shown in Table 1, the proportion of oxygen vacancies within the WO_3_-3 h-400 (20.1%) significantly exceeded those of WO_3_-3 h-600 (16.8%) and WO_3_-3 h-800 (14.8%). This could be attributed to the weaker oxidation of the films at lower annealing temperatures and the concomitant limitation of grain growth, resulting in films with lower crystallinity, rendering the formation of oxygen vacancies more favorable [12,13]. In addition, the relative content of W^5+^ in a WO_3_ film gradually decreased as the annealing temperature increased (Table S2), further confirming that lower annealing temperatures were more favorable in generating oxygen vacancies. Oxygen vacancies are critical in gas-sensitive detection, as they not only serve as active sites to promote gas adsorption but also enhance the electron transport properties of the material [42,43]. Therefore, the higher oxygen vacancy content of the WO_3_-3 h-400 film improved the responsiveness of the gas sensor to 2-CEES, yielding a superior sensing performance.

## 5. Conclusions

In conclusion, WO_3_ porous thin films rich in oxygen vacancies were successfully fabricated via sputter deposition for the detection of the sulfur mustard simulant 2-CEES. The optimal process parameters were identified through systematic adjustments of the sputtering time and annealing temperature. Specifically, the WO_3_-3 h-400-based sensor exhibited a high response value (10.6) and rapid response (58 s) at 260 °C. Moreover, the sensor demonstrated an actual detection limit of 15 ppb, along with excellent repeatability, long-term stability over 30 days, and good selectivity. Based on the strong adsorption and charge transfer interaction between WO_3_ and 2-CEES, the controllable preparation of WO_3_ films with a porous structure and enhanced surface oxygen vacancies significantly improved the response performance of the sensors. Our work provides a potential strategy for the batch production of MOS-based miniaturized devices for real-time monitoring of vesicant gases.

## Figures and Tables

**Table 1:** Comparison of O 1s XPS spectra of WO_3_ thin films sputtered for 3 h under different calcination temperatures.

	O_L_	O_V_	O_ad_	O_V_/O_total_
WO_3_-3 h-400	530.39	531.55	532.77	20.1%
WO_3_-3 h-600	530.47	531.73	532.90	16.8%
WO_3_-3 h-800	530.40	531.72	532.72	14.8%

## Data Availability

Data are contained within the article.

## Supplementary Materials

The following supporting information can be downloaded at: https://www.mdpi.com/article/10.3390/s25103049/s1, Figure S1: Schematic illustration of an own-design gas sensing detection system; Figure S2: The standard curve of 2-CEES; Figure S3: XRD pattern of WO_3_ thin films prepared at different sputtering time and annealing temperature; Figure S4: SEM images of a pure Si/SiO_2_ substrate, (a) cross-sectional view and (b) top-view plane diagram; Figure S5: Temperature-dependent responses of the WO_3_-3 h-400-based sensor; Table S1: The sensing properties of the reported MOS-based 2-CEES gas sensors. Figure S6: AFM images of WO_3_ thin films at different sputtering times: (a) 10 min; (b) 30 min; (c) 1 h; (d) 2 h; (e) 3 h; Figure S7. SEM images of WO_3_ thin film annealed at 400 °C with different sputtering times: (a) 10 min; (b) 1 h; (c) 3 h; Table S2: Comparison of W 4f XPS spectra of WO_3_ thin films sputtered for 3 h under different calcination temperatures. References [3,7,44,45,46] are cited in Supplementary Materials.
