# Supplementary material for: Fabrication of Oxygen Vacancy-Rich WO3 Porous Thin Film by Sputter Deposition for Ultrasensitive Mustard-Gas Simulants Sensor
Source: Sensors (Basel). 2025 May 12;25(10):3049. doi: 10.3390/s25103049 (PMC12115028; doi:10.3390/s25103049)
Supplement: Supplementary file 1 [file sensors-25-03049-s001.zip › sensors-3595320-supplementary.pdf]

sensors MDPI

Supporting Information

Fabrication of oxygen vacancy-rich WO_3 porous thin film by sputter deposition for Ultrasensitive Mustard-Gas Simulants Sensor

Haizhen Li[1,2], Cancan Yan[2], Jun Shen[2], Shuai Liu[2], Qingyu Ma[1,*] and Yongchao Zheng[2,*]

[1] School of Materials Science and Engineering, University of Jinan, Jinan, 250022, China
[2] State Key Laboratory of Chemistry for NBC Hazards Protection, Beijing, 102205, P. R. China
* Correspondence: authors. E-mails: mse_maqy@ujn.edu.cn (Q. Y. Ma); zhengyongchao@mail.ipc.ac.cn (Y. C. Zheng)

Table of contents

1. Schematic illustration of an own-designed gas sensing detection system	Figure S1
2. The standard curve of 2-CEES	Figure S2
3. XRD pattern of WO_3 thin films	Figure S3
4. SEM images of a pure Si/SiO_2 substrate	Figure S4
5. Temperature-dependent responses of the WO_3-3 h-400-based sensor	Figure S5
6. The sensing properties of the reported MOS-based 2-CEES gas sensors	Table S1
7. AFM images of WO_3 thin films	Figure S6
8. SEM images of WO_3 thin film annealed at 400 °C with different sputtering times	Figure S7
9. Comparison of W 4f XPS spectra of WO_3 thin films	Table S2

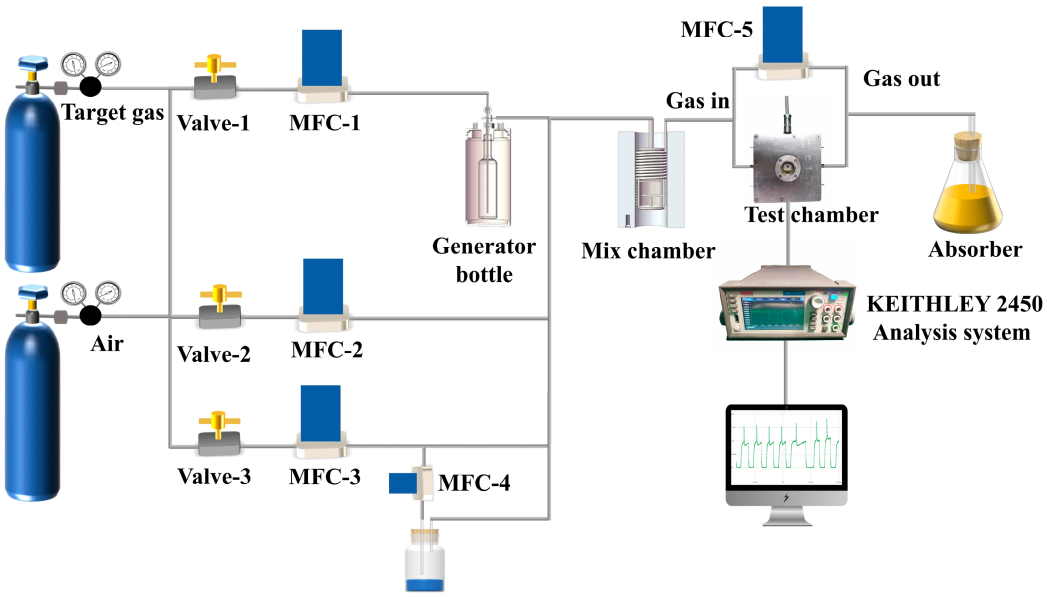

Figure S1. Schematic illustration of an own-designed gas sensing detection system. MFC: mass flow controller.

The gas sensing measurements were carried out using the custom-designed gas sensing detection system, as illustrated in Figure S1. The maximum total gas flow rate of the system can reach 2 L min^{-1}, and the mass flow controller (MFC-1) can stably control the gas flow of 0.02-0.04 ml min^{-1}, which ensures the occurrence concentration of the ppb level. The mass flow controller (MFC-5) is used to divert gas flow, which ensures the consistency of test conditions (intake volume) when detecting low-concentration targets. The saturated vapor pressure of 2-CEES is 3.79 mmHg at 25 °C, and it is easy to condense at room temperature. To ensure the stability of the system after the occurrence of the target object, the gas path is set to a constant temperature of 120 °C to effectively avoid the instability of the detection system caused by changes in the external environment.

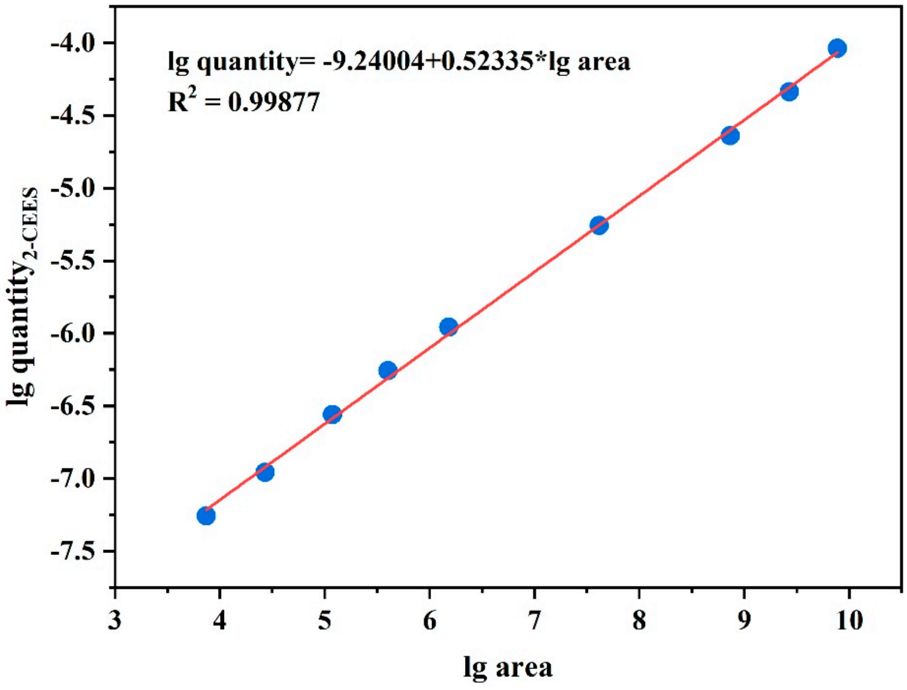

Figure S2. The standard curve of 2-CEES.

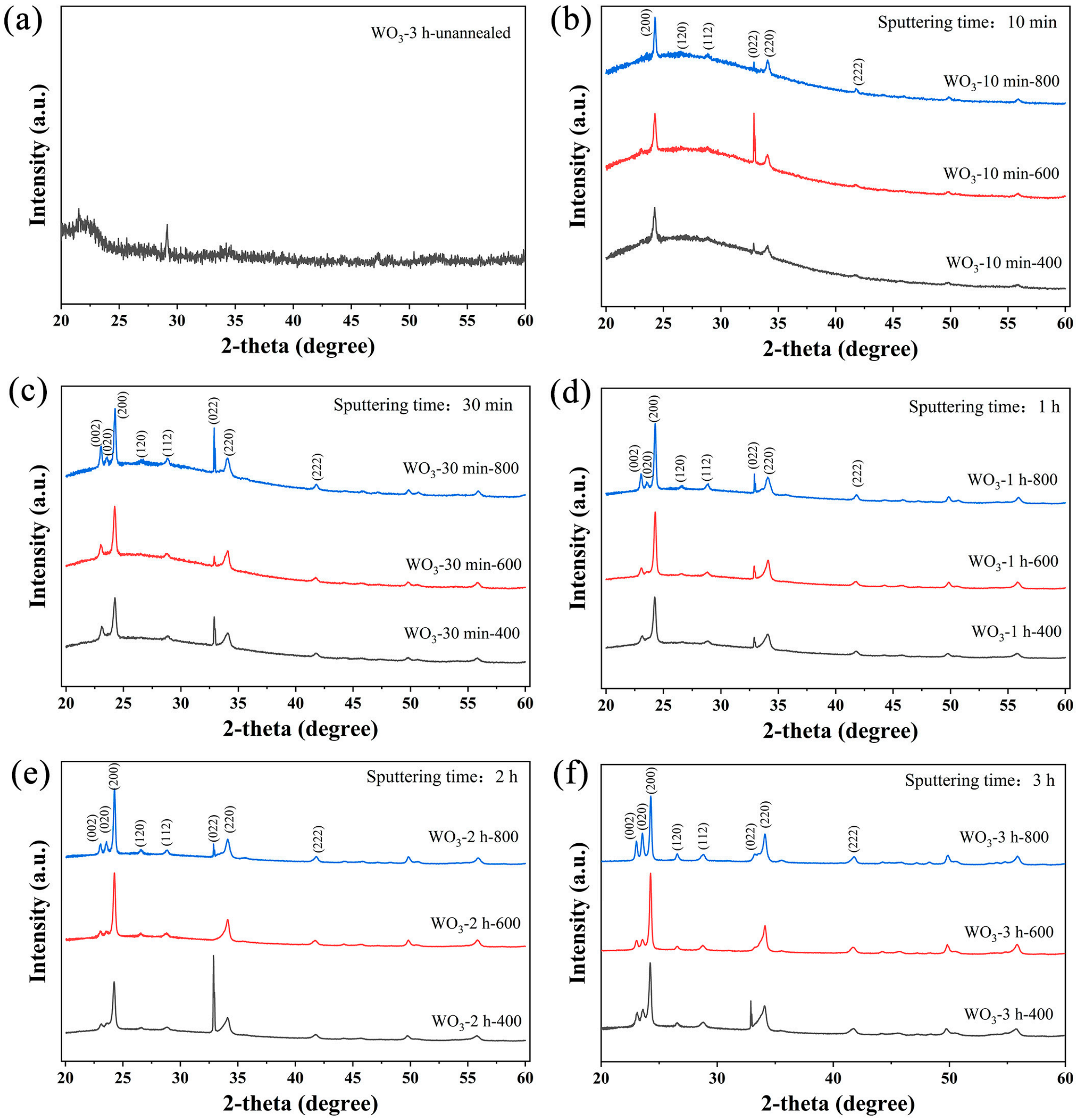

Figure S3. XRD pattern of WO_3 thin films prepared at different sputtering times and annealing temperatures.

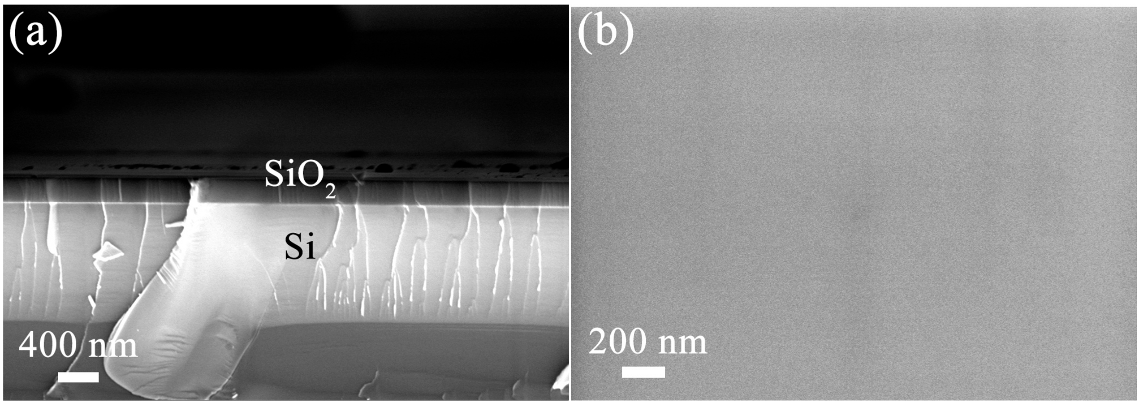

Figure S4. SEM images of a pure Si/SiO_2 substrate, (a) cross-sectional view and (b) top-view plane diagram.

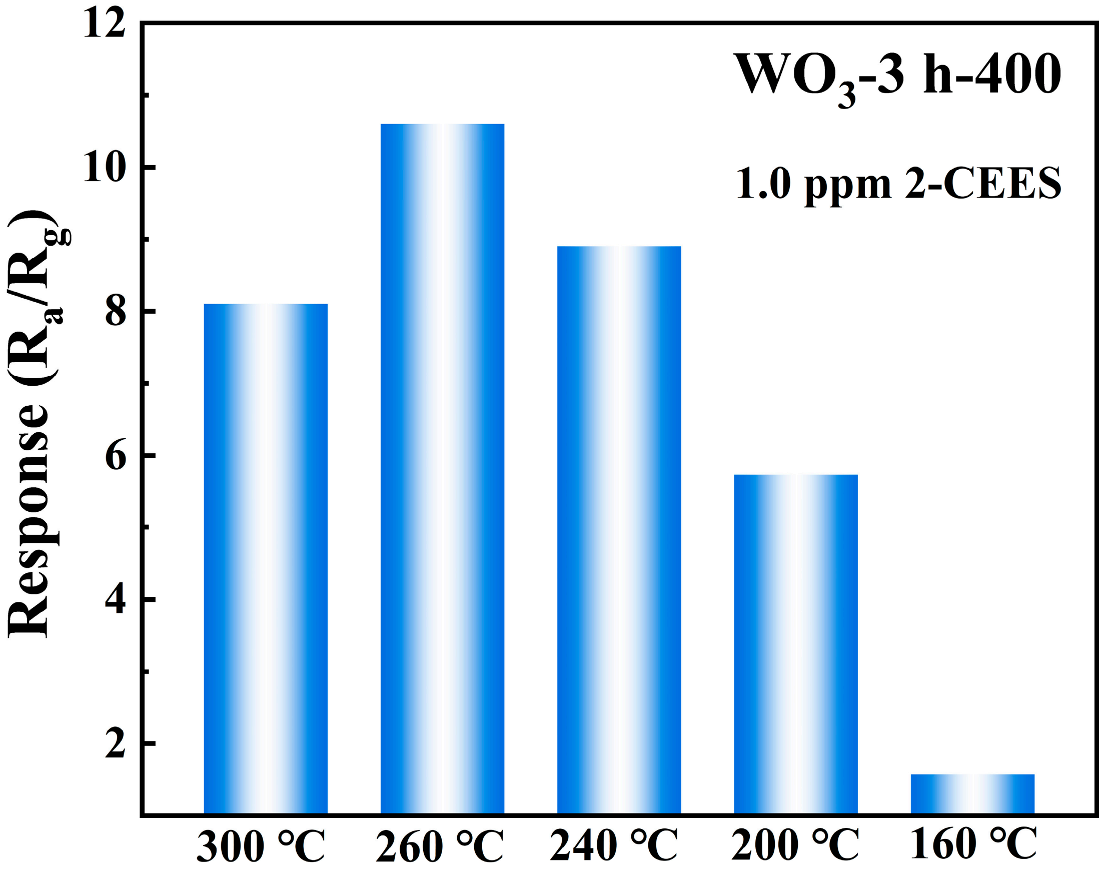

Figure S5. Temperature-dependent responses of the WO_3-3 h-400-based sensor toward 1.0 ppm 2-CEES.

Table S1. The sensing properties of the reported MOS-based 2-CEES gas sensors.

**Material**	**Preparation method**	**Operating temperature (◦C)**	**Concentration (ppm)**	**Response value**	**Sensitivity (ppm^{-1})**	**Practical limit of detection (ppm)**	**Ref**
ZnO thick film	Ultrasonic atomization	400 °C	2	3	1.5	-	[1]
$CdSnO_3$	Ultrasonic spray pyrolysis	500 °C	4	12	3	0.5	[2]
Core–shell $ZnFe_2O_4$ microspheres	Solvothermal	250 °C	1	9.07	9.07	0.1	[3]
WO_3 nanonet	In situ solvothermal	217 °C	50	58	1.16	0.3	[4]
Fe_2O_3 nanotube array	In situ solvothermal	170 °C	10	9.6	0.96	0.03	[5]
WO_3-3 h-400	Sputter deposition	260 °C	1.0	10.6	10.6	0.015	This work

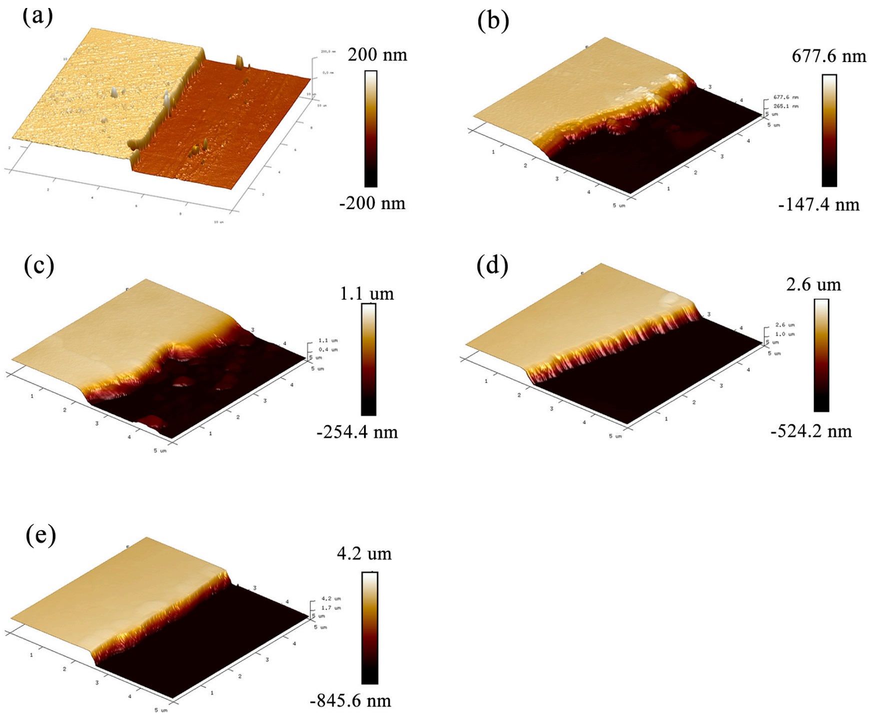

Figure S6. AFM images of WO_3 thin films at different sputtering times: (a) 10 min; (b) 30 min; (c) 1 h; (d) 2 h; (e) 3 h.

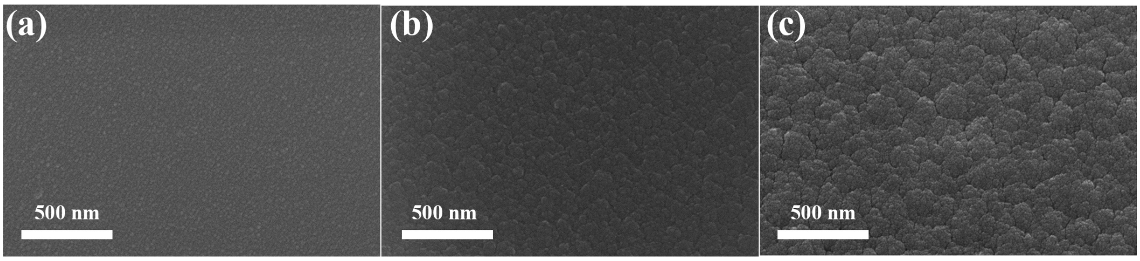

Figure S7. SEM images of WO_3 thin film annealed at 400 °C with different sputtering times: (a) 10 minutes; (b) 1 hour; (c) 3 hours.

Table S2. Comparison of W 4f XPS spectra of WO_3 thin films sputtered for 3 h under different calcination temperatures.

	$W^{6+}4f_{7/2}$	$W^{6+}4f_{5/2}$	$W^{5+}4f_{7/2}$	$W^{5+}4f_{5/2}$	W^{6+}/W^{5+}
WO_3-3 h-400	34.54	36.61	35.55	37.68	1:0.16
WO_3-3 h-600	34.58	36.67	35.59	37.72	1:0.14
WO_3-3 h-800	34.60	36.72	35.66	37.79	1:0.12

References

1. Patil, L. A.; Bari, A. R.; Shinde, M. D.; Deo, V.; Kaushik, M. P., Detection of dimethyl methyl phosphonate – a simulant of sarin: The highly toxic chemical warfare – using platinum activated nanocrystalline ZnO thick films. *Sens. Actuators, B* **2012,** 161, (1), 372-380.
2. Patil, L. A.; Deo, V. V.; Shinde, M. D.; Bari, A. R.; Patil, D. M.; Kaushik, M. P., Ultrasonically Sprayed Nanostructured Perovskite-Type $CdSnO_3$ Thin Films for Sensing of CWA Simulants. *IEEE Sens. J.* **2014,** 14, (9), 3014-3020.
3. Yang, J.; Yang, L.; Cao, S.; Yang, J.; Yan, C.; Zhang, L.; Huang, Q.; Zhao, J., High-performance metal-oxide gas sensors based on hierarchical core-shell ZnFe(2)O(4) microspheres for detecting 2-chloroethyl ethyl sulfide. *Anal Methods* **2023,** 15, (25), 3084-3091.
4. Zheng, Q.; Wang, T.; Li, B.; Gao, R.; Zhang, X.; Cheng, X.; Huo, L.; Major, Z.; Xu, Y., Crosslinked WO_3 nanonet for rapid detection of sulfur mustard gas simulant: Mechanism insights and sensing application. *Sens. Actuators, B* **2023,** 385.
5. Li, B.; Ma, X.; Xin, Y.; Major, Z.; Zhang, X.; Wang, T.; Huo, L.; Cheng, X.; Xu, Y., In situ construction of hierarchical Fe_2O_3 nanotube arrays for real-time detection and degradation of 2-CEES gas. *Sens. Actuators, B* **2023,** 383.
